# Ultrasound‐Based SECURED Protocol for Central Venous Catheterization: A Feasibility Study

**DOI:** 10.1155/bmri/9445119

**Published:** 2026-06-19

**Authors:** Moon Soo Choi, Han Ho Do, Jeong Hun Lee, Seung Chul Lee, Sanghun Lee

**Affiliations:** ^1^ Department of Emergency Medicine, Dongguk University Ilsan Hospital, Goyang, Republic of Korea, dongguk.edu

## Abstract

**Background:**

Central venous catheterization (CVC) is essential for managing critically ill patients but is associated with potential mechanical complications. Although ultrasound‐guided (USG) CVC is widely used, its application is often fragmented, focusing primarily on needle guidance. We developed and evaluated the “Standardized Evaluation of Central venous catheterization using Ultrasound for Risk Elimination and Detection” (SECURED) protocol, which integrates validated point‐of‐care ultrasound (POCUS) techniques into a unified, six‐step sequential workflow.

**Methods:**

This prospective observational study enrolled 109 adult patients undergoing internal jugular vein (IJV) CVC in the emergency department (ED). The SECURED protocol consists (1) Pre‐CVC IJV scan, (2) Pre‐CVC pleural scan, (3) USG IJV puncture, (4) Guidewire insertion and confirmation, (5) Post‐CVC pleural scan, and (6) Post‐CVC cardiac scan with microbubbles. The primary endpoint was the protocol completion rate, whereas the secondary endpoint was a comparison of the time required for ultrasound‐based complication detection versus portable chest radiography.

**Results:**

The SECURED protocol achieved a 100% completion rate across all 109 cases. The protocol successfully identified one case of preexisting IJV thrombosis, prevented the progression of two arterial punctures, and detected one venous dissection during the procedure. Furthermore, the protocol enabled rapid detection of post‐CVC complications, identifying one pneumothorax and two catheter tip malpositions. The median time required to confirm pneumothorax and catheter tip malposition using the SECURED complication‐detection steps was significantly shorter than that with chest radiography (2.5 min [95% CI: 2.3–2.8] vs. 12.4 min [95% CI: 11.1–13.7]; *p* < 0.001).

**Conclusions:**

The SECURED protocol is a practical and highly time‐efficient screening framework that bridges the gap between fragmented ultrasound adjuncts and standardized clinical practice. By enabling proactive hazard detection and immediate postprocedural confirmation, it serves as a feasible screening tool that streamlines clinical decision‐making in the ED. Following further validation through larger, multicenter studies with comparative designs to evaluate its clinical efficacy and safety benefits, broader implementation of this protocol across various clinical settings could be considered.

## 1. Introduction

Central venous catheterization (CVC) is an essential procedure performed in the emergency department (ED) and intensive care unit for hemodynamic monitoring and the administration of vasoactive drugs, fluids, and blood products [1]. Despite its critical role, CVC is associated with mechanical complications, including vascular injury, pneumothorax, hemothorax, thrombosis, and catheter malposition [1–4]. The traditional landmark‐based method for CVC has been linked to complication rates as high as 15%, with the potential for these rates to double with repeated attempts [1, 4, 5]. Consequently, ultrasound‐guided (USG) CVC has become the standard of care, significantly reducing both immediate mechanical complications and catheter‐related bloodstream infections [3–7]. Although ultrasound guidance is well recognized for improving success rates and reducing complications [2–7], it does not completely eliminate the risk of arterial puncture or vascular injury [8, 9].

Current clinical guidelines strongly advocate for the use of point‐of‐care ultrasound (POCUS) primarily to facilitate real‐time needle guidance and preprocedural vessel assessment [8–11]. Although various postprocedural applications of POCUS—such as pleural scanning for pneumothorax detection and microbubble transit time assessments for catheter tip localization—are well documented in the literature [12–16], these safety measures are typically implemented as disconnected, optional adjuncts rather than a unified standard of care.

Accordingly, we developed the “Standardized Evaluation of Central venous catheterization using Ultrasound for Risk Elimination and Detection” (SECURED) protocol. The novelty of the SECURED protocol lies in its systematic integration of these validated POCUS techniques into a standardized six‐step sequential workflow. Unlike existing bundles that focus predominantly on the insertion phase, the SECURED protocol establishes a comprehensive evaluation pathway that encompasses the entire procedure, from initial vessel selection to final confirmation. By defining the precise timing and sequence for each ultrasound intervention, the protocol ensures that potential hazards are proactively identified at every critical transition point rather than being detected post hoc.

We hypothesized that this logical synchronization would facilitate efficient bedside screening and the early identification of procedural hazards. To evaluate this hypothesis, we conducted a study to assess the feasibility and clinical utility of the SECURED protocol in adult patients undergoing internal jugular vein (IJV) CVC in the ED. To validate the protocol′s diagnostic performance, all participants underwent postprocedural chest radiography as a comparative standard. We specifically investigated the occurrence of procedural complications and evaluated the time‐efficiency of their detection using the SECURED protocol relative to conventional radiographic imaging.

## 2. Materials and Methods

A prospective observational study was conducted from March 2020 to February 2021 in the ED of a university hospital with an annual patient volume of approximately 40,000 visits. The study enrolled patients aged ≥ 18 years with consent who required CVC. Patients were excluded if they were aged < 18 years, presented with cardiac arrest or chest trauma, underwent CVC at a different site, declined participation, or had incomplete medical records. As this was an exploratory feasibility study, no formal power calculation was performed; instead, a convenience sample from the defined study period was used. The study protocol was approved by the hospital’s institutional review board (IRB No. 2020‐01‐040).

**Figure 1:**
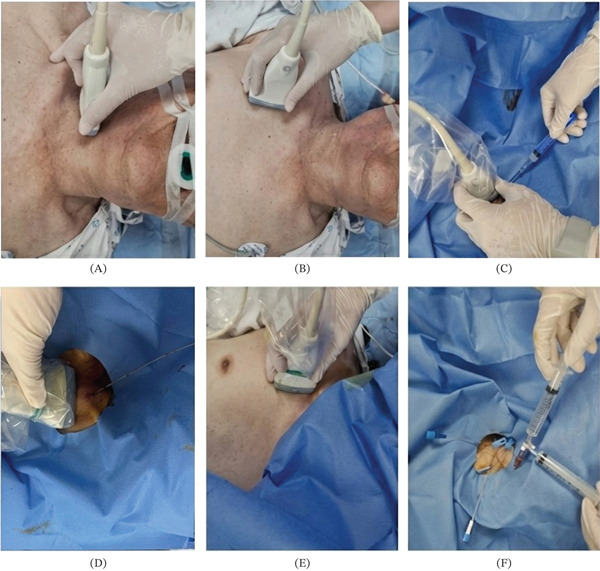
Six steps of the SECURED protocol. (A) Pre‐CVC IJV scan. (B) Pre‐CVC pleural scan. (C) USG IJV puncture. (D) Guidewire insertion and confirmation. (E) Post‐CVC pleural scan. (F) Post‐CVC cardiac scan with microbubbles.

The SECURED protocol consists of six sequential steps based on ultrasound examination during CVC (Figure 1). A detailed checklist is provided in Table 1.

• Step 1 (Pre‐CVC IJV scan): Assess the IJV for thrombus, stenosis, or anatomical anomalies. If any abnormality is detected, the procedure via the ipsilateral vessel should be abandoned. Additionally, if the IJV lies anterior to the carotid artery, the patient′s head rotation is adjusted to reposition the vein laterally, thereby optimizing the anatomical conditions for safe catheterization.
• Step 2 (Pre‐CVC pleural scan): Evaluate the ipsilateral pleura for the presence of lung sliding sign or lung pulse sign.
• Step 3 (USG IJV puncture): Perform real‐time visualization of needle entry during IJV puncture to minimize the risk of vascular injury.
• Step 4 (Guidewire insertion and confirmation): Insert the guidewire and, under ultrasound guidance, confirm its presence within the IJV before dilation; if not visualized, the procedure via the ipsilateral vessel is abandoned.
• Step 5 (Post‐CVC pleural scan): Reassess the pleura for pneumothorax, indicated by the abolition of lung sliding sign.
• Step 6 (Post‐CVC cardiac scan with microbubbles): Inject microbubbles via the inserted CVC while performing a cardiac ultrasound to confirm their rapid appearance in the right atrium. (within 1 s).

**Table 1:** Checklist of the SECURED protocol.

SECURED protocol		
Step 1 Pre‐CVC IJV scan	□ Suitable for CVC	□ Completed □ Failed scan
	□ Unsuitable for CVC	
	If any abnormality is detected, the procedure via the ipsilateral vessel should be abandoned. If the vein lies anterior to the artery, adjust head rotation to reposition the vein lateral to the artery.	

Step 2 Pre‐CVC pleural scan	Pleural attachment signs: □ Present □ Absent	□ Completed □ Failed scan
	Check the ipsilateral lung for pleural movement before the procedure. If lung sliding sign is absent, check for lung pulse sign.	

Step 3 USG IJV puncture	Needle tip visualization	□ Completed □ Failed scan
	□ Confirmed □ Not visualized □ Arterial injury	
	Perform USG needle insertion into the IJV. Confirm whether the needle has entered the artery or vein. If arterial entry occurs, immediately withdraw the needle from the artery and reposition it within the venous lumen under real‐time ultrasound guidance to safely continue the procedure. Apply 5‐min manual compression to the puncture site at the end of the procedure.	

Step 4 Guidewire insertion and confirmation	Guidewire visualization in IJV: □ Confirmed □ Not confirmed	□ Completed □ Failed scan
	Insert the guidewire under ultrasound guidance and scan the proximal IJV above the clavicle for confirmation. If the guidewire is not visualized, the procedure via the ipsilateral vessel should be abandoned, and the vessel should be evaluated for potential injuries or misplacement.	

Step 5 Post‐CVC pleural scan	Reassess pleural attachment signs: □ Present □ Absent	□ Completed □ Failed scan
	Reassess pleural movement after the procedure. The abolition of the pleural movement is highly suggestive of pneumothorax, necessitating immediate preparation for chest tube insertion.	

Step 6 Post‐CVC cardiac scan with microbubbles	□ Push‐to‐bubble time < 1 s	□ Completed □ Failed scan
	□ Push‐to‐bubble time > 1 s	
	Administer agitated saline while scanning the right heart. If microbubbles appear within 1 s, the catheter tip is considered correctly positioned. If transit time exceeds 1 s, do not use the catheter until its position is confirmed by chest radiography.	

Abbreviations: CVC, central venous catheterization; IJV, internal jugular vein; SECURED, Standardized Evaluation of Central venous catheterization using Ultrasound for Risk Elimination and Detection; USG, ultrasound‐guided.

Ultrasound images were obtained at each step, with pleural movements and microbubble appearances recorded as video clips. The procedures were performed by four emergency medicine (EM) residents who had completed a basic POCUS training course hosted by the Society of Emergency and Critical Care Imaging (SECCI), a domestic academic society recommended by the Korean Society of Emergency Medicine (KSEM). This basic POCUS training consisted of a 2‐h session on CVC and a 6‐h session on cardiopulmonary ultrasound. After completing the SECCI POCUS course, the participating residents had performed at least 20 cases each of USG CVC and cardiopulmonary POCUS independently. The principal investigator (PI) was an EM specialist with 15 years of experience in performing and teaching POCUS. All scans were conducted using a LOGIQ S8 ultrasound system (General Electric Healthcare, Chicago, Illinois, United States) equipped with a 2.5–8.0 MHz linear transducer and a 1.5–5.0 MHz phased array cardiac transducer.

Patient demographics, procedure times (including the duration of each step and the order‐to‐confirmation time of chest radiography for verifying catheter placement and detecting complications), CVC‐related complications, and anatomical site of the SECURED application were recorded. To minimize detection and confirmation bias, procedural step durations were measured by a nonparticipating PI. The PI determined each step′s duration by analyzing the timestamps of ultrasound data recorded throughout the SECURED protocol. Additionally, chest radiography turnaround times were systematically recorded.

The primary endpoint was the completion rate of the SECURED protocol steps. Successful completion was operationally defined as the definitive sonographic identification of target structures at each step, encompassing both normal anatomy and abnormal findings. Notably, cases in which the procedure was discontinued or modified due to the detection of abnormalities were still categorized as successful completions. This classification reflects the protocol′s core objective of proactive hazard detection, where the early identification of potential complications is considered a pivotal clinical outcome for mitigating procedural risk.

The secondary endpoint was a comparison between the incremental time required for the SECURED complication‐detection steps and the turnaround time of chest radiography. To isolate the specific time burden of our protocol, we calculated the duration of the SECURED complication‐detection steps (Steps 2, 5, and 6), excluding the mandatory steps of standard USG CVC (Steps 1, 3, and 4). This was then compared against the total turnaround time of chest radiography, measured from the initial order to final image confirmation. This comparison aimed to assess the procedural efficiency of the SECURED protocol, contextually analyzing its incremental time investment relative to the systemic turnaround delays inherent in conventional radiographic verification.

Data normality was assessed using the Shapiro–Wilk test (*p* < 0.05 defining nonnormality). Accordingly, continuous variables are reported as mean ± standard deviation (SD) or median (interquartile range, IQR) with 95% confidence intervals (CIs); for the latter, CIs were calculated via a rank‐based nonparametric method. Categorical variables are presented as frequencies and percentages (%). Between‐group comparisons of procedural time were performed using the Mann–Whitney *U* test. Effect sizes were reported as the rank‐biserial correlation (*r*). Statistical significance was defined as a two‐sided *p* < 0.05. All analyses were performed using IBM SPSS Statistics for Windows, Version 20.0 (IBM Corp., Armonk, New York, United States).

## 3. Results and Discussion

**Figure 2:**
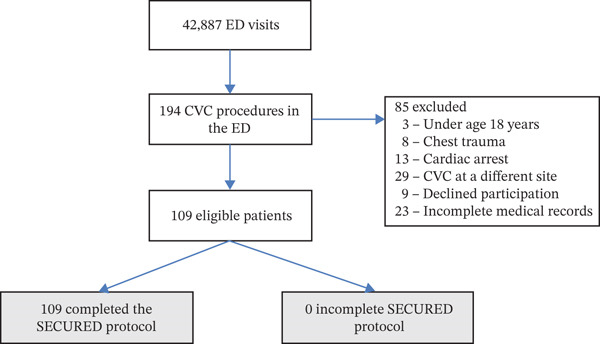
Flowchart of the study. ED, emergency department; CVC, central venous catheterization; SECURED, Standardized Evaluation of Central venous catheterization using Ultrasound for Risk Elimination and Detection.

In total, 109 patients were enrolled in the study (Figure 2). The mean age was 64.5 years, and 67 (61.5%) patients were male. The SECURED protocol was performed via the right IJV in 105 (96.3%) cases. The baseline characteristics are presented in Table 2.

**Table 2:** Baseline characteristics of the participants.

Characteristic	Value
Total patients	109
Age (years), mean ± SD	64.5 ± 15.4
Male sex, *n* (%)	67 (61.5%)
BMI > 25, *n* (%)	42 (38.5%)
Anatomical site of the SECURED application	
Right IJV, *n* (%)	105 (96.3%)
Left IJV, *n* (%)	4 (3.7%)
Indication for central catheter insertion	
Administration of specific agent, *n* (%)	67 (61.5%)
Hemodynamic monitoring, *n* (%)	29 (26.6%)
Massive transfusion/volume resuscitation, *n* (%)	9 (8.3%)
Poor peripheral venous access, *n* (%)	4 (3.7%)
Outcome of SECURED protocol	
Quit procedure before catheterization, *n* (%)	2 (1.8%)
Maintained catheter with proper position, *n* (%)	105 (96.3%)
Removed catheter due to malposition, *n* (%)	2 (1.8%)
Findings	
IJV thrombosis (Step 1), *n* (%)	1 (0.9%)
Arterial puncture (Step 3), *n* (%)	2 (1.8%)
Venous dissection (Step 4), *n* (%)	1 (0.9%)
Pneumothorax (Step 5), *n* (%)	1 (0.9%)
Catheter malposition (Step 6), *n* (%)	2 (1.8%)

Abbreviations: BMI, body mass index; IJV, internal jugular vein; SD, standard deviation; SECURED, Standardized Evaluation of Central venous catheterization using Ultrasound for Risk Elimination and Detection.

**Figure 3:**
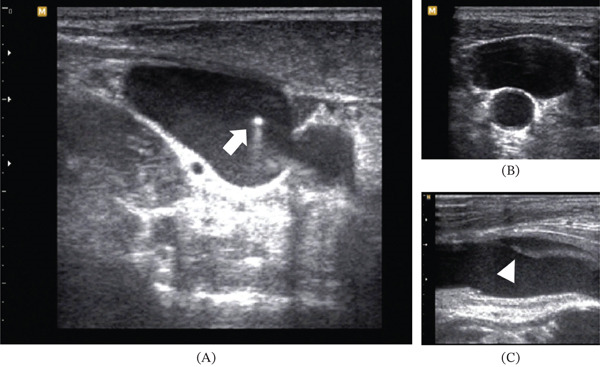
Sonographic confirmation of the guidewire in the IJV. (A) Visible guidewire (arrow) with a comet‐tail artifact below. (B) No visible guidewire inside the vein in this patient. (C) Jugular vein dissection (arrowhead) observed in the same patient.

**Figure 4:**
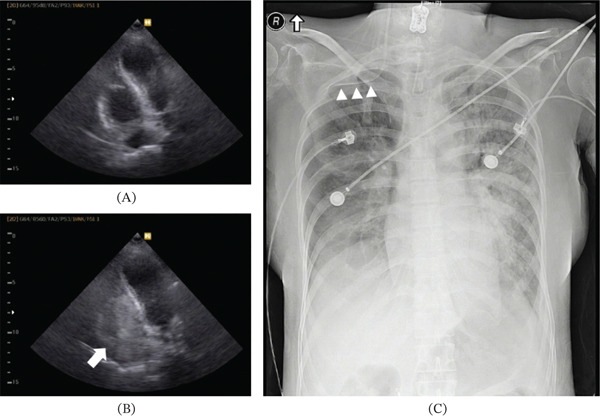
Postprocedural cardiac ultrasound exam with agitated saline. (A) Apical four‐chamber view. (B) Microbubble detected in the right heart 2.5 s after injection (arrow). (C) Catheter tip malposition (arrowhead) confirmed via chest radiography in the same patient.

The SECURED protocol was attempted in all 109 patients and completed in 100% of cases (Table 3, Figure 2). In Step 1, an IJV thrombus was identified in 1 of 109 patients, leading to protocol‐defined termination of the procedure on the affected side. In Step 2, lung sliding sign was confirmed in all 108 remaining patients. In Step 3, the needle tip was visualized in all 108 patients. Although arterial wall puncture occurred in two cases, it was immediately recognized. The needle was quickly repositioned within the IJV under ultrasound guidance, allowing the procedure to successfully continue in all 108 patients. In Step 4, the proximal IJV was successfully imaged in all 108 patients, and the guidewire was visualized within the IJV in 107; in the remaining patient, an IJV dissection was identified and the procedure was discontinued (Figure 3). In Step 5, pleural movement was reassessed in 107 patients; in one patient, the disappearance of the previously observed lung sliding sign was identified in 41 s, prompting a presumptive diagnosis of procedure‐related pneumothorax that was subsequently confirmed on chest radiography. In Step 6, the cardiac scan was successfully performed in 107 patients. In two cases, push‐to‐bubble transit time exceeded 1 s, with bubble visualization achieved at 2.5 and 4 s, respectively. These findings indicated catheter malposition, which was subsequently confirmed by chest radiography (Figure 4). Ultimately, all 109 patients completed the SECURED protocol. CVC was successfully performed in 107 of these patients, and the central venous catheter was maintained in position in 105 patients (96.3%).

**Table 3:** Completion rate of each step.

SECURED protocol attempted (*n* = 109)					
	Total	Completed		Failed	Completion rate
		Normal	Abnormal		
Step 1: Pre‐CVC IJV scan	109	108	1	0	100%
Step 2: Pre‐CVC pleural scan	108	108	0	0	100%
Step 3: USG IJV puncture	108	106	2	0	100%
Step 4: Guidewire insertion and confirmation	108	107	1	0	100%
Step 5: Post‐CVC pleural scan	107	106	1	0	100%
Step 6: Post‐CVC cardiac scan	107	105	2	0	100%

Abbreviations: CVC, central venous catheterization; IJV, internal jugular vein; SECURED, Standardized Evaluation of Central venous catheterization using Ultrasound for Risk Elimination and Detection; USG, ultrasound‐guided.

The total time required to complete the entire six‐step SECURED protocol had a median of 652 s (IQR: 552–759; 95% CIs: 609–679) (Table 4). The cumulative time for the three SECURED complication‐detection steps (Steps 2, 5, and 6) had a median of only 149 s (IQR: 114–180: 95% CIs: 137–166), whereas the turnaround time for portable chest radiography, measured from initial order to final image confirmation, had a median of 743 s (IQR: 568–902; 95% CIs: 664–820). The Mann–Whitney *U* test indicated that this difference was highly significant (*U* = 18, *Z* = −12.6, *p* < 0.001), with a large effect size (rank‐biserial, *r* = 0.86).

**Table 4:** Time consumption of each step in the SECURED protocol and portable radiography.

	N	Median [IQR] (seconds)	95% CIs for Median (seconds)
Step 1: Pre‐CVC IJV scan	109	28 [22–39]	26–31
Step 2: Pre‐CVC pleural scan	108	42 [33–52]	37–45
Step 3: USG IJV puncture	108	182 [161–222]	173–191
Step 4: Guidewire confirmation	108	272 [204–362]	235–292
Step 5: Post‐CVC pleural scan	107	32 [24–43]	30–36
Step 6: Post‐CVC cardiac scan	107	63 [42–101]	53–74
SECURED protocol total (Step 1–6)	107	652 [552–759]	609–679
SECURED complication‐detection steps (Step 2, 5, 6)	107	149 [114–180]	137–166
Chest radiography (order to image acquisition)	107	743 [568–902]	664–820

Abbreviations: CIs, confidence intervals; CVC, central venous catheterization; IJV, internal jugular vein; IQR, interquartile range; SECURED, Standardized Evaluation of Central venous catheterization using Ultrasound for Risk Elimination and Detection; USG, ultrasound‐guided.

This study demonstrates the feasibility of the SECURED protocol, a systematic six‐step ultrasound workflow for CVC in the ED. By addressing the clinical gap left by traditional disconnected ultrasound adjuncts, our research establishes a unified end‐to‐end framework that encompasses the entire procedural spectrum—from initial vessel assessment to final confirmation. This sequential approach facilitates real‐time decision‐making and early hazard detection, allowing clinicians to interrupt the progression of procedural risks before they manifest as overt complications.

Conducting a preprocedural vascular assessment is crucial for ensuring patient safety, particularly given the high prevalence of anatomical variations in the IJV. Thrombosis, stenosis, and structural deformities may be present even in patients without a history of neck surgery or radiation [17, 18]. Additionally, neck rotation or posture can alter the anatomical relationship between the IJV and internal carotid artery; notably, excessive rotation may position the vein anterior to the artery, increasing the risk of arterial puncture [19]. In our study, the preprocedural scan (Step 1) identified an IJV thrombus in one patient, prompting immediate termination of the procedure. This initial assessment not only optimizes procedural efficiency by precluding unnecessary sterile preparation for unsuitable vessels but also establishes the anatomical foundation for a safer intervention. Studies emphasize that precise needle tip visualization is indispensable for avoiding posterior wall puncture of the IJV, particularly in cases where the vein is compressed [20, 21]. To proactively address this risk, the SECURED protocol utilizes Step 1 to optimize patient positioning, such as adjusting head rotation to reposition the vein and minimize vascular overlap. By proactively creating these ideal anatomical conditions, the protocol facilitates the rigorous real‐time needle visualization required in Step 3, thereby substantially reducing the risk of inadvertent posterior wall injury. The precision of this visualization can be further enhanced by employing technical adjuncts, such as echogenic or marked introducer needles. For instance, Ghatak et al. [22, 23] demonstrated that the use of marked needles significantly improves needle tip identification. Incorporating such evidence‐based tools could provide an additional procedural safeguard to the SECURED protocol, particularly for less experienced operators who may encounter difficulties in needle‐vessel alignment. Mandatory guidewire confirmation (Step 4) was included before dilation because sonographic verification of the guidewire within the venous lumen markedly reduces the risk of unintentional arterial dilation [24]. Despite two instances of arterial puncture, immediate recognition and needle repositioning prevented progression to accidental guidewire insertion or arterial dilation; this proactive intervention averted potentially severe vascular trauma, with no subsequent complications observed. Using the supraclavicular scanning technique described by Maheshwari et al. [25], which involves visualizing the IJV superior to the clavicle and proximal to the puncture site, we confirmed guidewire placement in 107 patients. In one case in which the guidewire was not visible, additional scanning revealed an IJV dissection, resulting in immediate cessation of the procedure.

Pneumothorax is a significant complication that requires prompt evaluation following CVC. In patients receiving mechanical ventilation, pneumothorax can rapidly lead to severe hemodynamic instability, making early diagnosis essential. Pleural integrity can be confirmed by identifying lung sliding sign or lung pulse sign, both of which are definitive markers of pleural apposition. Lung sliding sign represents the visceral pleura gliding against the parietal pleura during respiration, whereas lung pulse reflects transmitted cardiac vibrations in the absence of respiratory movement. The presence of either sign ensures that the ultrasound beam has reached the visceral pleura without air interference, effectively ruling out pneumothorax. Several studies have indicated that confirming the lung sliding sign through an anterior chest ultrasound scan can exclude pneumothorax with a 99% negative predictive value [26–28]. Thus, the presence of the lung sliding sign after the procedure provides strong evidence that pneumothorax has not occurred. Conversely, the absence of this sign may suggest pneumothorax, although it can also result from pleural adhesions or atelectasis [28]. Therefore, diagnosing pneumothorax based solely on the absence of the lung sliding sign after the procedure has inherent limitations. To address this, the SECURED protocol employs a comparative approach by assessing pleural movement both before (Step 2) and after (Step 5) the procedure. The development of a new abolition of pleural movement at the same anatomical site is highly specific for pneumothorax. Using this method, we accurately diagnosed one case of pneumothorax, which was subsequently confirmed by chest radiography. Because postprocedural chest radiography was performed for all patients, we verified that no cases of pneumothorax were missed by the SECURED protocol.

Verifying the position of the central catheter tip is a critical component of the SECURED protocol, particularly in Step 6. This verification is performed by measuring the time interval between the administration of agitated saline microbubbles through the catheter and their subsequent visualization in the right atrium. According to Ley et al. [29], a push‐to‐bubble transit time of ≤ 1 s is a reliable indicator of correct catheter tip placement. In our study, prolonged bubble transit times accurately identified two cases of catheter malposition, with transit times of 2.5 and 4 s, respectively. These findings were later confirmed by chest radiography, which demonstrated catheter positioning within the subclavian vein.

As demonstrated in our findings, the diagnostic component of the SECURED protocol (Steps 2, 5, and 6) was completed with a minimal time investment, offering immediate point‐of‐care evaluation. This efficiency arises from the seamless integration of these steps into the procedural workflow, providing rapid diagnostic feedback independently of the multi‐step processes required for conventional radiography. Notably, our protocol′s performance (median: 149 s) is shorter than the 5‐minute confirmation window previously reported by Duran‐Gehring et al. [30]. This superior speed is likely attributable to the continuous and proactive design of the workflow; as the ultrasound is already operational, subsequent diagnostic steps are executed without the setup delays. Furthermore, the preprocedural preparation likely facilitates more rapid image acquisition during the cardiac scan in Step 6, reinforcing the protocol′s clinical utility in time‐sensitive ED settings.

However, this study has some limitations. First, this was an exploratory, single‐center study utilizing a convenience sample without a formal sample size calculation. Although a sample size of 109 cases is sufficient to validate the procedural workflow and technical feasibility of the SECURED protocol, the use of convenience sampling may introduce selection bias and limit the statistical power to detect rare mechanical complications or draw definitive comparative conclusions. Furthermore, specific exclusion criteria may limit the generalizability of our findings. Patients in active cardiac arrest were excluded to prioritize standardized resuscitation and minimize nonessential procedural delays, although those who achieved return of spontaneous circulation (ROSC) were included. Additionally, patients with chest trauma were excluded because pre‐existing traumatic injuries could act as confounding variables, potentially obscuring the relationship between the CVC and complications such as pneumothorax. Second, interoperator variability was not explicitly evaluated. Nevertheless, based on previous literature [31–33], we anticipated that the protocol could be successfully implemented by residents with basic POCUS training, which was subsequently confirmed by our results. Furthermore, this study focused primarily on the feasibility and systematic flow of the SECURED protocol, rather than assessing individual performance variations. Third, small or occult pneumothorax might have been missed, as neither ultrasound nor chest radiography is as sensitive as computed tomography (CT) for detecting minimal pneumothorax. In this study, routine CT was not performed, as exposing asymptomatic patients to additional radiation solely to identify minimal pneumothorax was deemed clinically and ethically unjustified. Although chest radiography was performed on all patients postprocedure to identify complications such as pneumothorax and catheter tip malposition, the immediate timing of the radiography may have failed to detect small or occult pneumothoraces that had not yet fully manifested. Consequently, the actual incidence of procedure‐related pneumothorax might have been underestimated. Fourth, there is a potential for detection or confirmation bias regarding procedural turnaround times. To minimize this, step durations were strictly measured via retrospective review of saved video clips by an independent investigator using standardized and rigorous criteria for the start and end points of each step. Additionally, regarding the diagnostic performance of the SECURED protocol, all step‐by‐step abnormal findings (such as the abolition of lung sliding sign or delayed bubble transit time) were evaluated and documented immediately after the procedure. This approach rendered these interpretations independent of and blinded to the subsequent portable chest radiography results. Nevertheless, since the performing residents were not blinded to the clinical context, the possibility of performance bias (specifically the Hawthorne effect) and operator interpretation bias cannot be entirely excluded; operators might have performed the protocol more rapidly than in routine clinical practice. Furthermore, a formal third‐party blinded adjudication of the ultrasound images was not performed. Fifth, our comparison of turnaround times between the SECURED protocol and portable chest radiography did not fully account for the inherent systemic workflow constraints of radiography, such as equipment transport, image processing, and interpretation. These systemic factors differ fundamentally from the bedside nature of POCUS. Therefore, the significantly shorter time to detection observed with the SECURED protocol should not be interpreted as a claim of absolute clinical superiority over conventional radiography. Instead, it highlights the protocol′s operational value as a proactive screening framework that provides immediate clinical feedback to emergency physicians, thereby narrowing the diagnostic latency period during the critical postprocedural window before radiographic confirmation. Finally, the absolute frequency of complications observed in this study was extremely low (e.g., pneumothorax: *n* = 1, catheter malposition: *n* = 2). Furthermore, due to the lack of a comparative design, the direct impact of the SECURED protocol on complication reduction could not be determined. Therefore, these findings should be interpreted as descriptive observations within a feasibility framework, and future controlled, comparative studies are warranted to rigorously evaluate the protocol′s efficacy in reducing complications.

## 4. Conclusions

In conclusion, the SECURED protocol offers a structured evolution in CVC workflows by integrating fragmented POCUS evidence into a standardized sequential framework. Beyond the needle‐insertion phase emphasized in current guidelines, its novelty lies in the seamless sequential integration of preprocedural screening and postprocedural complication detection. Our findings confirm that this comprehensive framework is both feasible and operationally efficient within the demanding ED environment, facilitating rapid, real‐time clinical decision‐making. Although large‐scale multicenter randomized trials with comparative designs are warranted to definitively establish its risk‐mitigation efficacy, this protocol holds promise as a valuable bedside tool to optimize procedural workflows and support clinical risk management.

## Funding

This work was supported by the Dongguk University, 10.13039/501100002471.

## Conflicts of Interest

The authors declare no conflicts of interest.

## Data Availability

The data that support the findings of this study are available from the corresponding author upon reasonable request.
